# Assessment of the Synergic Effect between *Lysinibacillus sphaericus* S-Layer Protein and Glyphosate in the Lethality of the Invasive Arboviral Vector *Aedes albopictus*

**DOI:** 10.3390/insects11110793

**Published:** 2020-11-12

**Authors:** Mario Dániel-Gómez, Jenny Dussán

**Affiliations:** Microbiological Research Center (CIMIC), Department of Biological Sciences, Universidad de Los Andes, Bogotá 111711, Colombia; mf.daniel10@uniandes.edu.co

**Keywords:** *Lysinibacillus sphaericus*, S-Layer, glyphosate, *Aedes albopictus*, synergy

## Abstract

**Simple Summary:**

The tiger mosquito is a novel vector for a variety of viral diseases in Colombia. Glyphosate herbicides have been extensively used in the country as a means to battle illicit crops, namely coca. Negative effects of this compound on arthropods have been reported, but no emphasis on dipterans has been evaluated. Different bacilli bacteria, including the Colombian *Lysinibacillus sphaericus*, have shown mosquitocidal potential through the production of different proteins. The surface layer (S-Layer) protein, present in this bacterial species, is involved in normal processes, such as protection and shape, but it has been reported as having a role in the mosquitocidal action of the species. In this paper, we evaluate the toxicity of glyphosate, its derivates, and the bacterial S-Layer protein on tiger mosquito larvae, as well as the synergic effect these compounds may have. Bacterial-derived formulations may provide an alternative to chemical pest control and be a viable way to remediate environmental contamination consequences of the drug war.

**Abstract:**

Glyphosate and glyphosate-based herbicides are among the most used chemicals in plant pest control. Both glyphosate and its main by-product Aminomethylphosphonic Acid (AMPA) are highly environmentally persistent and, through several processes (including surface runoff and bioaccumulation), affect species beyond their intended targets, especially in aquatic ecosystems. *Aedes albopictus* is a novel invasive arboviral vector in Colombia and has spread to much of the national territory in recent years. Strains of the bacterium *Lysinibacillus sphaericus* have shown the ability to degrade glyphosate into environmentally inert compounds, in addition to having great larvicidal efficiency in different mosquito species through the production of several proteins, including the surface layer (S-Layer) protein. The S-Layer is a bacterial structure consisting of glycoprotein monomers, and its functions are thought to include bacterial interactions, protection from the outside medium and biological control. The study assessed the entomopathogenic activity of *L. sphaericus* S-Layer protein on *Ae. albopictus* larvae, and the effects that glyphosate and its by-products have in this process. To that end, bioassays were performed to compare the larval mortality between different treatments with and without S-Layer, glyphosate, and glyphosate derivates. Comparisons were made through Analysis of variance (ANOVA) and Tukey’s Honestly Significant Difference (HSD) analyses. Significant differences were found in larval mortality in the treatments, and larval mortality was greater when the S-Layer protein was present, though glyphosate field-doses (1.69 g/L) alone had a notable toxicity as well. An apparent synergic effect on the mortality of larval *Ae. albopictus* when exposed to mixtures containing 1500 ppm of the S-Layer protein, glyphosate, and/or glyphosate derivates was found. Further studies are needed for the in-depth understanding of this mechanism and its consequences on aquatic ecosystems.

## 1. Introduction

Glyphosate is one of the most widely used herbicides around the world, as it has shown to be useful in the eradication of a multitude of plant pests [1]. This, combined with its wide availability and the development of glyphosate-resistant crops, has made it into a desirable control strategy for crop protection, as well as activities like gardening and flower trade [2]. However, recent studies have questioned the safety of glyphosate and glyphosate-based herbicides, as they have shown an array of risks for animals and microorganisms alike, ranging from antimicrobial activity to mammal death at acute concentrations [3,4]. Moreover, it possesses the ability to bind to soil molecules and bioaccumulate in some organisms [5,6]. This confers it a notable persistence in the environment, in turn extending its ability to cause harm [5]. In recent decades, political and economic circumstances have led to the widespread dispersion of glyphosate throughout the Colombian countryside [7,8]. Thus, the failing efforts to control the production of coca plants, and in turn cocaine, have caused the endangerment of non-target species across different realms in the biosphere [3,4,9]. Furthermore, given how surface runoff displaces chemicals and materials present in soil towards water bodies [10], aquatic-dwelling organisms are especially susceptible to glyphosate and its decay products [11].

*Aedes* (Stegomyia) mosquitoes are the most important arboviral vectors in Colombian public health today, as they can carry a multitude of different diseases [12]. The recent invasion by a novel *Aedes* mosquito, *Aedes albopictus* (Skuse) [13] is a matter of concern because of the rising mosquito resistance to chemical control [14] and the negative impact these chemicals have on the environment [15]. Because of these reasons, novel control strategies have been devised in order to control the spread of high mortality and morbidity diseases such as Dengue [16]. Given how early mosquito developmental stages occur in water bodies [17] and the fact that they have shown greater susceptibility towards both chemical and biological control means [18], there exists a real need to evaluate the impact that toxic contaminants like glyphosate may have over mosquito populations. This, in turn, can help shed light on how glyphosate contamination works in an aquatic medium and raise concerns over the use of this substance and its consequences on non-target species.

The bacterium *Lysinibacillus sphaericus* is a widely common soil bacterium, and it has been found living in Colombian territory [19]. Strains of this bacterium are not only effective in mosquito control but have also shown potential as a metal and hydrocarbon bioremediation agents [20,21], as well as plant growth promoters [22]. Though studies have been conducted on several of the mosquitocidal pathways present in *L. sphaericus* strains [23,24], the roles that proteins such as the surface layer (S-Layer) protein and other derivates from the vegetative [25] *L. sphaericus* cell play in mosquitocidal action are open to further study, as their mechanisms and roles have not been explained in full. Though the most widely produced by-product of glyphosate mineralization is the highly toxic Aminomethylphosphonic Acid (AMPA) [26], previous studies have shown that *L. sphaericus* is able to degrade glyphosate into less dangerous compounds (orthophosphate ion and glycine, which are non-toxic) through the sarcosine oxidase metabolic pathway [26,27]. This raises the need to not only evaluate the role of glyphosate itself, but also the role its by-products may have in possible synergistic effects when in contact with this bacterium and its proteins. In this study, we aim to evaluate the lethality induced by the S-Layer protein on *Ae. albopictus* larvae, as well as the synergy that may occur in glyphosate and glyphosate derivate-rich environments, such as the Colombian countryside.

## 2. Materials and Methods

The methodology employed and outlined below is a modification of the protocol utilized previously by Bernal and Dussán in 2020 and Lozano et al. in 2011 [8,23].

### 2.1. Lysinibacillus sphaericus and Aedes albopictus Strains

The *L. sphaericus* III(3)7 strain was isolated from an oak forest soil in Colombia [19]. The WHO reference strain 2362 was isolated from adult *Simulium damnosum* [28] and kindly donated by A. Delecluse from the Pasteur Institute in France. These bacterial strains were chosen for the S-Layer protein extraction and posterior assays, as they have shown the most lethality in *Ae. aegypti* mosquitoes among the *L. sphaericus* strains present in the Microbiological Research Center (CIMIC) collection, having an LD50 concentration of 10^7^ CFU/mL [16]. The eggs of the Colombian reference *Ae. albopictus* strain were kindly donated by the National Institute of Health (INS) in Bogotá, Colombia.

### 2.2. S-Layer Protein Extraction, Purification, and Quantification

Selected bacterial strains were grown overnight in nutrient broth and under constant stirring (150 rpm at 30 °C). Overnight cultures were centrifuged (9660 rcf at 4 °C) for 20 min, and the pellets were washed in cold 50 mM Tris/HCl (pH 7.4); the cells were broken by sonication (40% amplitude, 15 pulses) [29], washed three times and treated with 0.5% Triton X-100 for 10 min at 20 °C. The S-Layer protein was extracted using guanidine hydrochloride (Gu.HCl) (5 M in 50 mM Tris/HCl buffer, pH 7.4) for 4 h at 4 °C. After this step, samples underwent centrifugation at 11,337 rcf for 40 min at 4 °C. The supernatant containing the extracted S-Layer protein was dialyzed against Milli-Q (MQ) water at 4 °C for 20 h, centrifuged at 11,337 rcf for 40 min at 4 °C, and supernatants were stored at −20 °C. Supernatant samples were then recovered and quantified through the Bradford protein assay. A calibration curve was constructed using 20 μL of 0.125 mg, 0.25 mg, 0.5 mg, 0.75 mg, 1 mg, 1.5 mg and 2 mg of pure bovine albumin diluted in 70 μL of MQ water and then dyed with 1 mL of Bradford dye reagent. These were then measured at 595 nm in a spectrophotometer. With the calibration curve prepared, 20 μL of each of the supernatant samples recovered was assessed with 70 μL MQ water and 1 mL of Bradford dye reagent at 595 nm in the spectrophotometer. Confirmation of the presence of the S-Layer protein was assessed through SDS-PAGE protocol in a 10% acrylamide gel. Then, 6 μL samples of the S-Layer protein extracts were loaded in a 10% acrylamide gel and stained with Coomassie Brilliant Blue. The resulting gel was then cleaned with MQ water and revealed using a transilluminator. This protocol was followed for both bacterial strains, and solutions containing 1500 ppm (750 ppm per bacterial strain) of the S-Layer protein were made using MQ water to be used in the bioassays.

### 2.3. Glyphosate, Phosphate and Glycine Solutions

Standard 10 g/L solutions of each of the used compounds were prepared by diluting 10 g of solid pure glycine, monobasic potassium phosphate and Monsanto’s glyphosate formulation Roundup 747^®^ (Bayer, Leverkusen, Germany) in 1 L of MQ water. Solutions were then thoroughly stirred and kept in drawers to prevent decomposition due to UV light. These stock solutions were employed and diluted to the 1.69 g/L concentration used in the bioassays. This concentration was chosen as it is the most widely used in weed control by farmers, as well as the eradication of illicit crops like coca in Colombia [8]. Monobasic potassium phosphate was used as an orthophosphate ion source as it does not figure as a toxic agent for animals at the experimental concentrations, eliminating noise in the results [30].

### 2.4. Aedes Mosquito Maintenance and Bioassays of the S-Layer Protein, Glyphosate, Phosphate and Glycine against Aedes albopictus Larvae

Eggs donated by the INS were kept at 30 °C and 60–70% relative humidity under 12:12 light/dark photoperiod. Upon hatching, the larvae were fed with pellet food Omega One Natural Protein Formula (OmegaSea, LLC, Painesville, OH, USA) for cichlids, twice a week. The larvae were kept in plastic containers until the third instar was reached. In order to determine the possible synergic effects of glyphosate and glyphosate breakdown products (glycine and orthophosphate) with the bacterial S-Layer protein, several solution mixtures were made using the 1.69 g/L solutions. As mentioned before, this glyphosate concentration was chosen as it is the most widely used formulation in Colombia for pests and illicit crops control [8]. Equal concentrations were used for both glycine and phosphate, as the stoichiometry of glyphosate break up yields a 1:1 product/reactant ratio [26]. Previous studies into the mosquitocidal action of the S-Layer protein against *Culex quinquefasciatus* have shown significant results at concentrations as low as 500 ppm [26,31]. Given how comparative studies have pointed at *C. quinquefasciatus* being considerably more susceptible to *L. sphaericus* biocontrollers than *Aedes aegypti* [32], an experimental concentration of 1500 ppm of the S-Layer protein was chosen. The final concentration was achieved by mixing together 750 ppm of the extracted S-Layer protein from each of the *L. sphaericus* strains used (III(3)7 and 2362). The resulting mixtures were the following: S-Layer protein, glycine, phosphate, glyphosate, phosphate + glycine, S-Layer protein + glycine, S-Layer protein + phosphate, S-Layer protein + glyphosate and phosphate + glycine + S-Layer protein. Final treatment concentrations were set at 1.69 g/L, and those for the S-Layer proteins were set at 1500 ppm (Table S3). These were assessed against chlorine-free water as control. In order to assess the mosquitocidal potential, the tests were conducted in a total experimental volume of 30 mL, consisting of 15 mL of chlorine-free water and 15 mL of each of the solutions, into which 20 individuals were added. All trials were performed in triplicate. The number of live larvae was recorded every 24 h until 48 h.

### 2.5. Statistical Analysis

All statistical tests were performed using the R 3.1.2 statistical package [33], and a significance level of *p* < 0.05 was chosen for every test. Homoscedasticity was evaluated and confirmed through Bartlett’s test for homogeneity of variance, and data normality was assessed and verified through a Shapiro–Wilk normality test (Table S1). In order to assess statistically significant differences between mortality per treatment in the *Ae. albopictus* strain, analysis of variance (ANOVA) (one way) tests were performed followed by Tukey’s HSD tests as a post hoc analysis (Table S2).

## 3. Results

### 3.1. S-Layer Protein Extraction, Purification, and Quantification

**Figure 1:**
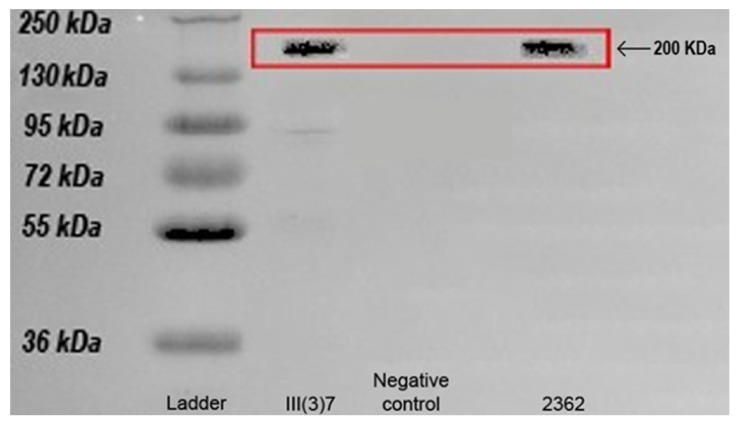
Acrylamide gel for the confirmation of surface layer (S-Layer) protein presence after the extraction protocol. The first lane corresponds to the protein ladder, the second ladder to *L. sphaericus* III(3)7 S-Layer protein, the third to a negative control treatment (MQ water), the fourth intentionally left empty, and fifth to *L. sphaericus* 2362 S-Layer protein.

The S-Layer protein was successfully extracted from the surface of both L. sphaericus strains. This was confirmed by the acrylamide gel and by comparing the obtained bands with both the protein ladder (Figure 1). The reported molecular weight of the protein assembly as the S-Layer is mainly composed of self-assembled proteins ranging in weight between 40–200 KDa [34]. Results indicate that both assembled the S-Layer protein, and S-Layer protein monomers were present in the L. sphaericus III(3)7 samples, while only assembled S-Layer protein structures were found in L. sphaericus 2362 samples. The extracted S-Layer protein concentrations varied between bacterial strains, with the 2362 strain having a concentration of 1.2 mg/mL, while the III(3)7 strain showed an extracted concentration of about 0.8 mg/mL. S-Layer protein extraction was successful for both bacterial strains, and concentrations were sufficient for further experimentation.

### 3.2. Glyphosate, Glyphosate Derivates and S-Layer Protein Induced Mortality in Ae. albopictus Mortality

**Figure 2:**
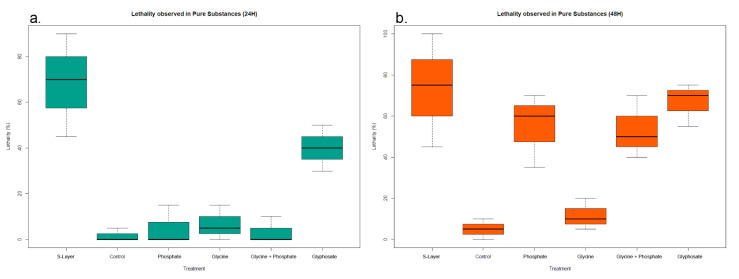
Lethality responses observed in the *Aedes albopictus* larvae after exposure to pure treatment solutions at (**a**) 24 h of exposure and (**b**) 48 h of exposure.

Larvicidal activity was registered past 24 and 48 h post inoculation. Results showed marked and significant (ANOVA: F(_5,12_) = 17.23, *p* < 0.0001) lethality differences between all treatments, especially considering larvae exposed to the S-Layer protein and glyphosate concentrations (Figure 2a). Significant differences were found when comparing active treatments with the control at 24 h for treatments containing glyphosate and the S-Layer protein, both having siginificant differences with every other treatment, except for each other. No significant differences were observed when comparing control, glycine and phosphate treatments among themselves (Table S2). Both glyphosate and S-Layer protein formulations showed an important mosquitocidal potential at 24 h, with the second having the most action against *Ae. albopictus* larvae in the lab environment. Past 48 h, a noticeable increase in larval lethality was observed in formulations containing orthophosphate ions, as well as a slight increase in glyphosate-induced lethality (Figure 2b). Larvicidal activity remained stable for both glycine and S-Layer protein formulations throughout the experiment. Significant differences between treatments were once more found past 48 h (ANOVA: F(_5,12_) = 9.902, *p* < 0.0001). Every treatment excluding glycine showed significant lethality differences when compared to the control, and no significant differences were observed when comparing glyphosate and phosphate with the S-Layer protein or each other (Table S2). Results obtained indicate an active role of the used compounds in mosquito larvae mortality.

### 3.3. Synergy between Glyphosate and Glyphosate Derivates with Bacteria S-Layer Protein in Ae. albopictus Mortality

**Figure 3:**
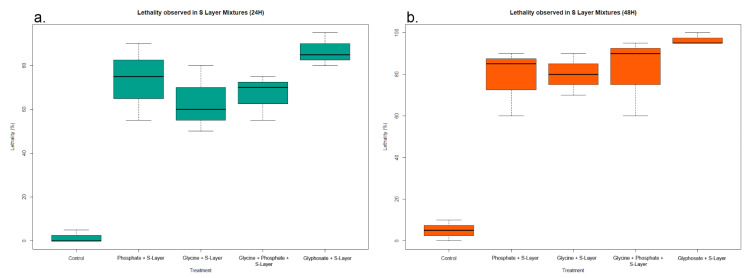
Lethality responses observed in the *Ae. albopictus* larvae after exposure to mixture of the treatment solutions and S-Layer concentrations at (**a**) 24 h of exposure and (**b**) 48 h of exposure.

Considerable larvicidal activity was observed throughout all the S-Layer protein mixture formulations at 24 h (Figure 3a). The greatest larval mortality was noted in formulations containing both glyphosate and the S-Layer protein. Significant differences were observed among all treatments (ANOVA: F(_4,10_) = 22.67, *p* < 0.0001), though no significant differences were found when comparing larvicidal action among the formulations containing the S-Layer protein, given the considerable mortality observed in them. Every single treatment besides the one containing both glycine and phosphate showed significant differences when compared to the control in this time frame (Table S2). The described trend remained constant past 48 h of exposure, and all formulations showed an increase in larvae mortality (Figure 3b). The highest larvicidal mortality was once again seen in formulations containing both glyphosate and the S-Layer protein. Significance was found when comparing between all treatments (ANOVA: F(_4,10_) = 26.14, *p* < 0.0001), as well as when comparing treatments containing the S-Layer to both the control and glycine treatments, but there was none between the treatments containing the S-Layer protein themselves (Table S2).

## 4. Discussion

### 4.1. S-Layer Protein Extraction, Purification, and Quantification

The S-Layer protein of both bacterial strains was successfully extracted, and further literature reviews clarified the fruitful extraction of the protein assembly [34]. The use and effectivity of the S-Layer protein as an insect controller has been proved in *C. quinquefasciatus* in the past [23,31], having notable mortality results. This study represents the second example in which the larvicidal potency of the S-Layer protein from *L. sphaericus* has been tested to satisfactory results in the target organism. Differences in extraction yields between strains are not derived from genetic differences, as both the WHO reference strain and the Colombian III(3)7 strain contain 13 S-Layer-related genes [35]. However, there may be differences in other transcription mechanisms regulating S-Layer gene transcription into S-Layer proteins in both strains, which could in turn lead to different protein production between them. Similarly, differences in the metabolic state between the bacterial strains do not pose a complete explanation for the differences observed. Studies conducted on *Bacillus anthracis*, another member of the Bacillaceae family, have found that the S-Layer proteins are also synthesized under conditions where the bacterial capsule is present, as it is an exterior layer and completely covers the S-Layer proteins [36]. Nonetheless, extraction assays performed using sporulated *L. sphaericus* cultures have failed to detect S-Layer protein presence by SDS-PAGE electrophoresis [23], which could indicate that the bacterial capsule acts in such a way that either denatures the S-Layer proteins or otherwise prevents their extraction and/or detection through SDS-PAGE assays. Though further experimentation is needed to clarify the nature of the difference observed, experimental or environmental influences cannot be crossed out as possible error or noise sources, especially considering the particularly complicated extraction protocol necessary for the extraction and purification of this protein.

### 4.2. Glyphosate, Glyphosate Derivates and S-Layer Protein Induced Mortality in Ae. albopictus Mortality

The role of field-dosed glyphosate concentrations in insect mortality has been shown through direct action in *Ae. aegypti* [8] and through indirect action through gut bacteria perturbation in *Apis mellifera* [37]. Additionally, a demographic study of *Chrysoperla externa* showed that glyphosate exposure had a significant impact on developmental stages, pre-reproductive periods, as well as fecundity and fertility, negatively impacting all of them. In addition, the chemical caused abnormal sizes and shapes in eggs, in addition to the appearance of tumors in the abdominal regions of adult individuals [38]. Results observed during the course of this investigation support previous findings and point at glyphosate as a damaging factor in environments and their dynamics. Additionally, common glyphosate derivatives such as AMPA have proven to be of even greater environmental concern, as AMPA in particular has greater soil persistence and equivalent toxicity in organisms [39]. Glyphosate derivatives employed in this study showed varying larval lethality percentages, as well as apparent time dependencies for their action. On one hand, the amino acid glycine showed little to no larval antagonism. This is not surprising, as glycine is used for the biosynthesis of many nonprotein compounds, such as porphyrins and purines, meaning its presence is paramount for normal animal development [40]. Although it has been suggested that since glycine plays a role in neurotransmitter regulation a high enough concentration could possibly have dangerous consequences [41], no evidence for animal toxicity was found for the concentrations used. On the other hand, phosphorus, another mineral needed in animal development [42], showed increasingly toxic trends in the larval assays as time went on. This may be linked to a decrease in water quality and oxygenation due to some form of eutrophication [43] or to the action of phosphate ions as acetylcholinesterase inhibitors, much like how organophosphates act [44]. Further studies are needed to clarify the role of orthophosphate ions in insect mortality.

The considerable mortality rates produced by the S-Layer protein against *Ae. albopictus* larvae mirror the results found of *C. quinquefasciatus* [23], meaning that it may be applied in the field as an effective mosquito controller to control a variety of vector-borne diseases. Furthermore, the data show that the use of living bacteria as biocontrol agents is not the only alternative as far as bacterial biocontrol goes, as metabolites such as the S-Layer protein can be used in their stead. Genome analyses of *L. sphaericus* have shown it is capable of producing a series of mosquitocidal toxins, as well as other proteins (e.g., hemolysin D) likely linked to its success as a mosquito control agent [45]. Metabolite-mediated mosquito control presents advantages to bacterial control, as proteins are environmentally inert, are not self-replicating, have no metabolic requirements and call for less intensive environmental studies (though the specificity of the toxin needs to be assessed, so as to prevent ecological damage to non-target species) before their application [46]. Despite their many advantages, the experiment only showed viable mosquitocidal action during the first 24 h. This likely stems from the protein denaturation brought about by factors like light intensity, UV radiation, pH, temperature, and animal activity [47]. Although it has been shown that bacterial S-Layer proteins are reasonably resistant to adverse conditions [48], it is likely that time and other factors reduced its efficiency considerably. In addition, protein-based biocontrol agents would carry several disadvantages, mainly the reduced environmental persistence brought about by the lack of environmental tolerance, plasticity, and recycling, as well as the lower tolerances for pH and temperature. This makes proteins significantly less environmentally persistent when compared to bacteria [49], which in turn means less mosquitocidal action per application when compared to bacterial biocontrollers [50]. Coupled with the above, the lack of automatic extraction procedures means significantly higher costs in both production and application, meaning further studies are needed before a viable biocontrol agent can be developed.

### 4.3. Synergy between Glyphosate and Glyphosate Derivates with Bacteria S-Layer Protein in Ae. albopictus Mortality

The effect of glyphosate and the lethality resulting from the synergy between glyphosate and vegetative *L. sphaericus* cells on *Ae. aegypti* has been established already [8]. The results found in this study support the hypothesis presented in the past, as there were significant increases in glyphosate lethality when paired with S-Layer bacterial extracts. However, the data show further synergy not only between the S-Layer protein and glyphosate, but its by-products as well, especially whenever orthophosphates are available. Mortality observed in glycine assays cannot be attributed to synergistic effects, as glycine showed to be non-toxic by itself. These results are of importance since *L. sphaericus* has been shown to degrade glyphosate into environmentally inert (and even beneficial) products [26,27]. The *L. sphaericus*-mediated release of orthophosphates and glycine from glyphosate into the environment may be a way to take advantage of a widely detrimental chemical and turn it into crop and plant growth promotion. This is even more likely considering the beneficial interactions seen between *L. sphaericus* and *Canavalia ensiformis* plants [22], though the indiscriminate release of orthophosphates to the environment may trigger detrimental ecological phenomena, such as eutrophication of water bodies and the resulting consequences of such events [43].

## 5. Conclusions

Ultimately, the use of either the bacterial S-Layer as a mosquito controller is a viable alternative to both traditional biocontrol and the more harmful chemical control. Positive synergic effects on the mortality of *Ae. albopictus* larvae after their exposure to the bacterial S-Layer, glyphosate and sarcosine pathway-generated glyphosate derivatives were observed, which prompts the support of the use of inert protein formulations for both vector control and the eradication of this invasive species. Further studies involving other *L. sphaericus* metabolites can help formulate even better biocontrol compounds. Similarly, more studies are needed for the in-depth comprehension of the ecological damages caused by glyphosate and related chemicals. The findings presented show the need for further investigation to maintain an appropriate balance between pest control (be it weed or insect) and proper ecosystem health.

## Supplementary Materials

The following are available online at https://www.mdpi.com/2075-4450/11/11/793/s1, Table S1: Statistical analyses of homoscedasticity and normality, Table S2: Post Hoc analysis of significance between treatments, Table S3: Treatments and concentrations used against *Aedes albopictus* larvae.
