# Supplementary material for: Assessment of the Synergic Effect between Lysinibacillus sphaericus S-Layer Protein and Glyphosate in the Lethality of the Invasive Arboviral Vector Aedes albopictus
Source: Insects. 2020 Nov 12;11(11):793. doi: 10.3390/insects11110793 (PMC7697419; doi:10.3390/insects11110793)
Supplement: Supplementary file 1 [file insects-11-00793-s001.zip › insects-972188-supplementary.docx]

**Table S1**. Statistical analyses of homoscedasticity and normality.

| **Shapiro-Wilk Normality Test** | |
| --- | --- |
| **Treatment (time)** | **p-value** |
| Pure treatment solutions (24 H) | 0.09 |
| Pure treatment solutions (48 H) | 0.2148 |
| Mixed treatment solutions (24 H) | 0.0828 |
| Mixed treatment solutions (48 H) | 0.1708 |

| **Bartlett's Test for Homogeneity of Variance** | |
| --- | --- |
| **Treatment (time)** | **p-value** |
| Pure treatment solutions (24 H) | 0.1965 |
| Pure treatment solutions (48 H) | 0.3507 |
| Mixed treatment solutions (24 H) | 0.3288 |
| Mixed treatment solutions (48 H) | 0.1902 |

**Table S2.** *Post Hoc* analysis of significance between treatments.

| **Tukey HSD (pure treatment solutions )** | | |
| --- | --- | --- |
| **Time** | **Treatment Comparison** | **p-value** |
| **24 H** | Control - S-Layer | <0.001 |
|  | Phosphate - S-Layer | <0.001 |
|  | Glycine - S-Layer | <0.001 |
|  | Control - Glyphosate | 0.014 |
|  | Glyphosate - Phosphate | 0.025 |
|  | Glyphosate - Glycine | 0.034 |
|  | Glyphosate - S-Layer | 0.08 |
|  | Control - Phosphate | 0.9 |
|  | Control - Glycine | 0.9 |
|  | Glycine - Phosphate | 0.9 |
| **48 H** | Control - S-Layer | 0.002 |
|  | Glycine - S-Layer | 0.004 |
|  | Control - Phosphate | 0.021 |
|  | Control - Glyphosate | 0.004 |
|  | Glycine - Phosphate | 0.0048 |
|  | Glyphosate - Glycine | 0.011 |
|  | Phosphate - S-Layer | 0.7 |
|  | Glyphosate - S-Layer | 0.9 |
|  | Control - Glycine | 0.9 |
|  | Glyphosate - Phosphate | 0.9 |

| **Tukey HSD Test (mixed treatment solutions )** | | |
| --- | --- | --- |
| **Time** | **Treatment Comparison** | **p-value** |
| **24 H** | Control - Phosphate + S-Layer | <0.001 |
|  | Control – Glycine + S-Layer | <0.001 |
|  | Control - Glycine + Phosphate + S-Layer | <0.001 |
|  | Control – Glyphosate + S-Layer | <0.001 |
|  | Glycine + Phosphate – S-Layer | <0.001 |
|  | Glycine + Phosphate – Glyphosate | 0.019 |
|  | Glycine + S-Layer – Glyphosate + S-Layer | 0.1 |
|  | Glycine + Phosphate + S-Layer – Glyphosate + S-Layer | 0.3 |
|  | Glyphosate + S-Layer – Phosphate + S-Layer | 0.6 |
|  | Glycine + S-Layer - Phosphate + S-Layer | 0.8 |
|  | Control - Glycine + Phosphate | 0.9 |
|  | Glycine + Phosphate - Phosphate | 0.9 |
|  | Glycine + Phosphate - Glycine | 0.9 |
|  | Glycine + Phosphate + S-Layer – Glycine + S-Layer | 0.9 |
|  | Glycine + Phosphate + S-Layer - Phosphate + S-Layer | 0.9 |
| **48 H** | Control – Phosphate + S-Layer | <0.001 |
|  | Control – Glycine + S-Layer | <0.001 |
|  | Control - Glycine + Phosphate + S-Layer | <0.001 |
|  | Control – Glyphosate + S-Layer | <0.001 |
|  | Glycine – Glycine + Phosphate | 0.004 |
|  | Control – Glycine + Phosphate | 0.026 |
|  | Glyphosate + S-Layer – Phosphate + S-Layer | 0.4 |
|  | Glycine + S-Layer – Glyphosate + S-Layer | 0.49 |
|  | Glycine + Phosphate + S-Layer – Glyphosate + S-Layer | 0.5 |
|  | Glycine + Phosphate – S-Layer | 0.6 |
|  | Glycine + Phosphate – Glyphosate | 0.8 |
|  | Glycine + Phosphate - Phosphate | 0.9 |
|  | Glycine + S-Layer - Phosphate + S-Layer | 0.9 |
|  | Glycine + Phosphate + S-Layer – Glycine + S-Layer | 0.9 |
|  | Glycine + Phosphate + S-Layer - Phosphate + S-Layer | 0.9 |

Table S3. Treatments and concentrations used against *Aedes albopictus* larvae*.*

| Treatment | Concentration (g/L) | S-Layer protein concentration (ppm) |
| --- | --- | --- |
| Control | - | - |
| S-Layer protein | - | 1500 |
| Glycine | 1.69 | - |
| Phosphate | 1.69 | - |
| Glyphosate | 1.69 | - |
| Glycine + Phosphate | 1.69 + 1.69 | - |
| Glycine + S-Layer protein | 1.69 | 1500 |
| Phosphate + S-Layer protein | 1.69 | 1500 |
| Glyphosate + S-Layer protein | 1.69 | 1500 |
| Glycine + Phosphate + S-Layer protein | 1.69 + 1.69 | 1500 |
